# *Streptococcus gallolyticus* infective endocarditis in a patient with systemic lupus erythematosus: a three-dimensional echocardiography evaluation

**DOI:** 10.1590/S1679-45082013000300020

**Published:** 2013

**Authors:** Rudyney Eduardo Uchôa de Azevedo, Ana Clara Tude Rodrigues, Lucas Arraes de França, Maria Luciana Zacarias Hannouche da Trindade, Marcelo Luiz Campos Vieira, Claudio Henrique Fischer, Samira Saady Morhy

**Affiliations:** 1 Hospital Israelita Albert Einstein, São Paulo, SP, Brazil.

**Keywords:** Lupus erythematosus, systemic, Endocarditis, *Streptococcus* Echocardiography/methods

## Abstract

A 42 year-old woman was referred to our hospital with a history of fever and poor general status for the last 30 days. She presented tachycardia and a systolic apical murmur. Laboratory tests revealed leukocytosis of 13,100/mL, hemoglobin of 8.4g/dL and positive systemic lupus erythematosus antibodies (anti-Ro/SSA, anti-La/SSB, anticardiolipin, and antinuclear antibodies); blood culture was positive for *Streptococcus gallolyticus*. Three-dimensional transesophageal echocardiography was performed and revealed multiple mitral valve vegetations, with leaflet perforation and important mitral regurgitation, as well as large aortic vegetation, with cusp perforation and severe regurgitation. Additionally, a small vegetation was observed on the tricuspid valve, which presented moderate regurgitation. Three-dimensional transesophageal echocardiography provides appropriate visualization of complications resulting from infectious endocarditis.

## RESUMO

Mulher de 42 anos foi encaminhada ao hospital com história de febre e queda do estado geral há 30 dias. À admissão, apresentava taquicardia e sopro sistólico na região apical. Os exames de laboratório mostraram leucocitose com 13.100/mL, hemoglobina 8,4g/dL e anticorpos positivos para lúpus eritematoso sistêmico (anti-Ro/SSA, anti-La/SSB, anticardiolipina e anticorpo antinuclear); hemocultura foi positiva para *Streptococcus gallolyticus*. Foi realizado um ecocardiograma transesofágico tridimensional, que mostrou múltiplas vegetações na valva mitral, com perfuração do folheto e refluxo importante, além de grande vegetação aórtica com perfuração valvar e refluxo importante adicionalmente. Pequena vegetação foi identificada na valva tricúspide, com um refluxo significativo. O emprego da ecocardiografia transesofágica tridimensional proporcionou o diagnóstico de complicações decorrentes de endocardite infecciosa.

## INTRODUCTION

Systemic lupus erythematous (SLE) is an inflammatory autoimmune disease with multiple organ involvement^(1)^. The natural history of the disease varies and is characterized by periods of remission and acute disease. Women, especially those between 20 and 30 years of age, are more affected than men.

The heart is frequently affected, specially the valves. Valvar lesions vary from mild thickening to large verrucouse lesions (Libman-Sacks endocarditis). The mitral valve is more frequently involved. Transesophageal ultrasound is a sensitive tool for diagnosis of valve impairment and can be used to determine the extension of complications and progression of valvar disease^(2)^.

*Streptococcus gallolyticus is a* Gram-positive coccus and is part of the *Streptococcus bovis* (*Streptococcus*) and *Streptococcus infantarius* group. It is an important cause of infectious bacteremia and endocarditis in adults. Infections by this agent were associated with colon tumors and gastrointestinal tract lesions^(3)^. However, the association between *Streptococcus gallolyticus* endocarditis and lupus causing valve leaflet perforation has not been described yet.

The three-dimensional echocardiography allows for multiplanar spatial observation of the heart and better understanding of the anatomy and geometry of cardiac structures. The use of three-dimensional echocardiography is recommended in the following situations: (1) to measure the volume of the left ventricle; (2) to determine the ejection fraction of the left ventricle; (3) to evaluate mitral valve anatomy; (4) to determine the degree of mitral valve stenosis; (5) to guide percutaneous procedures in the cath lab^(4)^. So far, three-dimensional echocardiography has not been extensively used for diagnostic investigation and identification of complications associated with infectious endocarditis^(4)^. In this case report three-dimensional transesophageal echocardiography was used in a SLE patient presenting with infectious endocarditis.

## CASE REPORT

**Figure 1:**
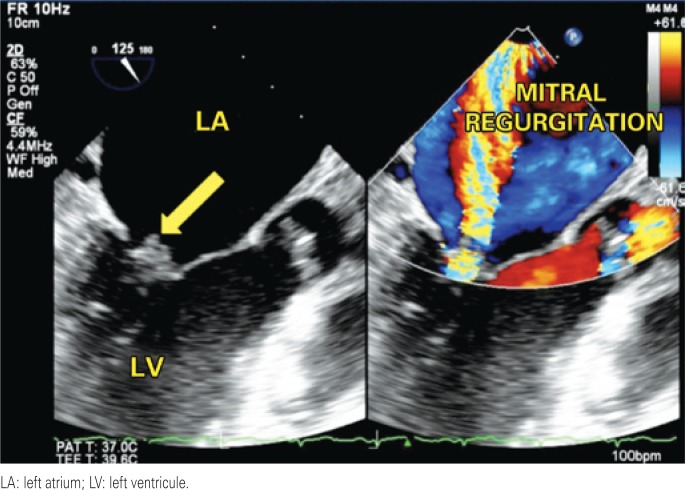
Two-dimensional transthoracic echocardiogram showing vegetation (arrow) and mitral regurgitation

**Figure 2:**
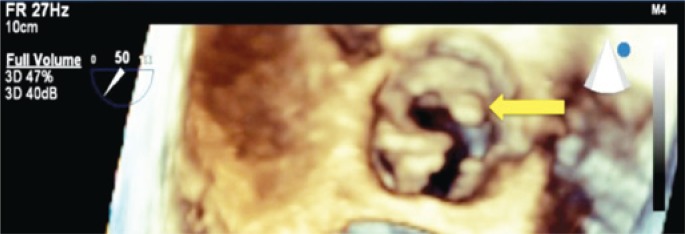
Three-dimensional transthoracic echocardiogram showing aortic leaflets thickness-systole (arrow)

**Figure 3:**
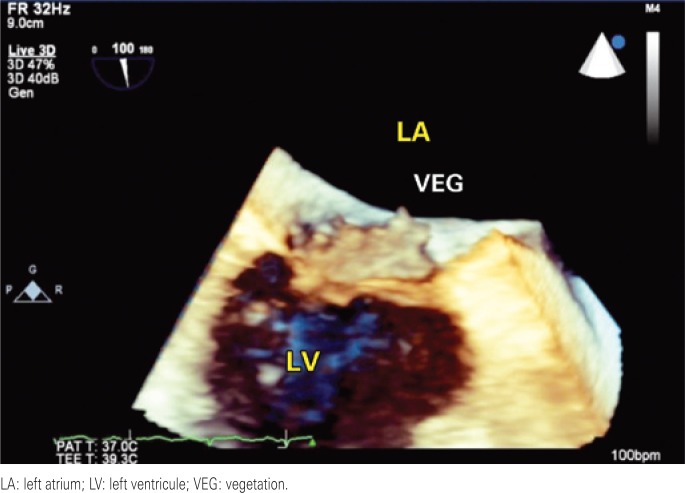
Three-dimensional transthoracic echocardiogram demonstrating mitral valve vegetation

**Figure 4:**
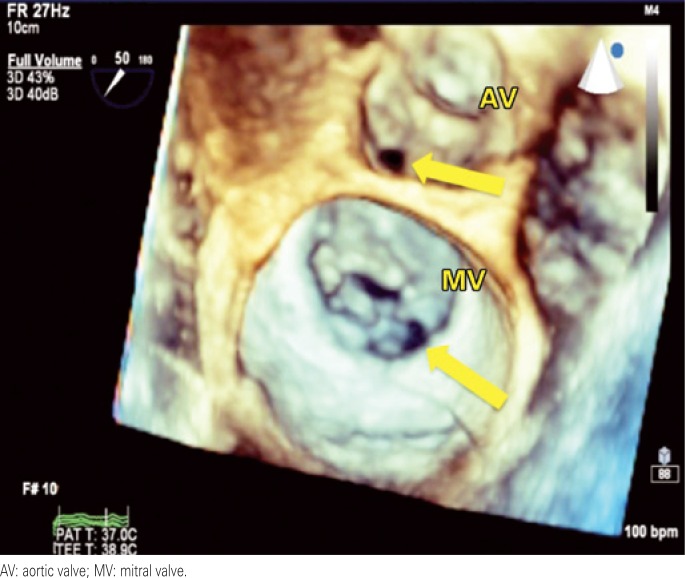
Three-dimensional transthoracic echocardiogram depicting mitral and aortic perforations (arrows)

A 42-year-old female patient was referred to our hospital with a history of fever and poor general status for 30 days. The physical examination upon admission presented axillary temperature 37.6°C; heart rate 110bpm; blood pressure: 120/80mmHg; and apical systolic murmur. Laboratory test results: 13,100 leucocytes/mL; hemoglobin 8.4g/dL; positive antibodies for SLE (anti-Ro/SSA, anti-La/SSB, anticardiolipin and anti-nuclear). Blood cultures tested positive for *Streptococcus gallolyticus*. Transthoracic echocardiogram revealed an image of a large aortic vegetation with mild regurgitation, and images of multiple vegetations on the mitral valve with severe regurgitation. Abdominal CT revealed multiple kidney, spleen and liver infarctions. Colonoscopy showed sigmoid diverticulum and two hyperplastic polyps. Histology of the colonoscopy findings suggested no evidence of colon neoplasia. Three days later, the bidimensional and three-dimensional transesophageal echocardiograms revealed multiple vegetations on the mitral valve, perforation of the cusp and important reflux and also an imaging of a great vegetation with valvular perforation. In addition, the exam also revealed a small vegetation on the tricuspid valve and severe regurgitation (Figure 1, 2, 3, 4). After four weeks of treatment, the patient's clinical picture worsened and she developed acute pulmonary edema, which required emergency surgical intervention. The intraoperative findings were compatible with destruction of the mitral and aortic apparatus. Surgical treatment consisted of mitral and aortic valve replacement and tricuspid valvuloplasty. The postoperative period was satisfactory, and she was discharged two weeks after surgery.

## DISCUSSION

Systemic lupus erythematous (SLE) is a pleomorphic condition that may pose a clinical challenge. In the case of this patient, the first manifestation of the disease was an infectious endocarditis, with multiple cardiac valve involvement, which is a very rare presentation. In addition, the isolation of *Streptococcus gallolyticus* from the blood was unexpected, because this agent causes important bacteremia and infectious endocarditis in elderly and adult men^(5,6)^. The presence of *Streptococcus gallolyticus* is frequently associated with lesions of the gastrointestinal tract, which were also present in this case. The fast destruction of the valves (perforation of two leaflets) is not common in patients with *Streptococcus gallolitycus* infection and is associated with increased morbidity and mortality^(6)^ .

Despite advances in medical and surgical treatments, infectious endocarditis still leads to high morbidity and mortality rates. Early identification of patients with large vegetations, as in the case described, is important to define the best therapeutic strategy, because, in these cases, surgical excision of the valve and valve replacement are followed by low perioperative mortality rates^(7)^.

In patients with endocarditis, the transesophageal echocardiogram is more suited for identification and determination of the degree of valve involvement than the transaortic echocardiogram. More recently, the three-dimensional echocardiogram added relevant pieces of information that contribute to determining the exact mechanism causing valve dysfunction. This is due to its higher spatial resolution and multi-angle visualization of the structures, which in the case described allowed for the exact determination of the site of leaflet perforation.
